# Epidemic Dysentery

**Published:** 1887-03

**Authors:** J. E. Wright

**Affiliations:** Columbia, La.


### Southern Medical Record.

A MONTHLY JOURNAL OF PRACTICAL MEDICINE.

☞All Communications must be addressed to R. C. Word, M. D., Managing Editor.

"*Quicquid Præcipies Esto Brevis.*"

Vol. XVII. ATLANTA, GA., MARCH, 1887. No. 3.

### ORIGINAL AND SELECTED ARTICLES.

### EPIDEMIC DYSENTERY.

By J. E. Wright, M. D., Columbia, La.

I propose to furnish you a brief account of an epidemic of malignant dysentery which prevailed in Caldwell and contiguous parishes, in the Summer of 1885. The scourge began the last week in May ; assumed epidemic proportions very soon ; culminated about the middle of July, and then began slowly to decline, both as to the number of cases and virulence of type. Fatal cases, however, occurred as late as the last of September and beginning of October. For deadly virulence and malignity of type it has, probably, had few paralells in modern times, especially amongst a rural population.

Few diseases, perhaps, known to our systems of nosological classification have attracted more attention, or had more able exponents than dysentery.

In the ordinary, as well as the purest classical descriptions, of this disease, the same name, with appropriate modifications, is used in all languages. Its classical history extends far back into remote antiquity. Nearly five thousand years before the Christian era, Heroditus tells of its terrible devastation of the great army of Xerxes in the plains of Thessaly ; Galen, A. D. 164, the great expositor of ancient medicine, has left on record a graphic description of the disease. So true is this, that the literature of the subject for many centuries after his time was little more than a paraphrase or amplification of his text. In more recent times, the semeiography of the disease has been so often and so ably treated by the great master minds of the profession that to dwell on that topic would be superfulous reiteration. The epidemic about to be described, however, had some distinctive features, so peculiar as to require special notice. The prodromic stage was short; the transition from perfect health to severe illness was often sudden. Nausea and vomiting often ushered in the attack; the vomited matter was usually dark green—sometimes almost black—thick and ropy in consistance, and very offensive in odor; uneasy sensations in the bowels; borborygmus eructations of gas, etc.; thirst and complete anorexia marked the inception of most cases. The alvine dejecta were diarrhœal in character at first, but soon became sero-sanguinolent. These, in turn, were soon succeeded by the mucous, blood, pus, shreds of pseudo-membranous matter, necrosed mucous tissue, etc., variously proportioned and commingled, and so characteristic of the disease in question as to be regarded by all observers as *pathognomonic.* At a still more advanced period of the disease the *lotura carnea* or fleshy stools, regarded since the time of Hippocrates as ominous of great danger, if not positively mortal, were occasionally seen. I cannot now recall to memory a single case in which these carneous stools appeared which recovered. One of the most noticeable of the objective features of this epidemic was the revolting odor of the dejecta. It was so pregnant, so all-pervading, and so unutterably disgusting as to defy description, yet never to be forgotten when once recognized. *Tormina* and *tenesmus*, usually so pronounced and troublesome in sporadic dysentery, were conspicuous only by their almost entire absence in this epidemic. The great and peculiarly distinctive feature of the scourge, however, was the adynamia—the utter prostration—which attended its very inception. From its first onslaught the aspect of the patient was suggestive of the most profound prostration of the physical energies. The countenance was pinched, shrunken, *Hippocratic*, ashy-pale, and cadaverous; the finger tip, pressed upon the surface and moved firmly on a line for an inch or two, leaves a blanched or white mark behind it, which very slowly regains the hue of the contiguous integument—thus distinctly suggesting the sluggishness, stagnation and almost complete stasis of the capillary circulation. A large superficial vein, being pressed upon so as to arrest the blood current, very slowly refills after the pressure is removed; the lips, tongue, buccal cavity, pharynx, etc., were leaden or livid in hue, and suggestive of cyanosis; the radical artery, a scarcely distinguishable thread, was obliterated by slight pressure; the pulse was usually accelated in frequency—often being 120 to 130 to the minute, and irregular in rhythm ; temperature but slightly elevated—often normal or sub-normal. This want of correlation between pulse and temperature was a marked feature throughout the entire course of the disease. The tongue was dry, contracted, and more or less coated with dark, foul-looking saburra, and cool or cold to the touch. The healthy hand laid flat on a patient's body received the sensation caused by the touch of a cold-blooded reptile ; the ganglionic centres of the nerves of organic life seemed to be paralyzed ; the functions of the vaso-motor and thermic centres were in almost complete abeyance, and the great central organ of the circulation profoundly embarrassed ; the respiratory movements were generally accelerated, and frequently interrupted by deep sighing ; restlessness, jactitation, insatiable thirst, and unspeakable disgust at the very thought of food were attendant in most cases throughout the entire course of the disease. Toward the close of some fatal cases complete paralysis of the sphincter ani muscle occurred, and the anal opening was sufficiently patulous to see the interior of the rectum several inches above the outlet. No case recovered in which this symptom occurred.

The experienced clinician who reads this array of symptoms will find obvious reasons for the appalling rate of mortality which attended the dreadful scourge. For the first three or four weeks the death-rate far exceeded that of the epidemic cholera, which was prevailing in Spain, France, and Italy at the time. I kept no mortuary record, but think it a moderate estimate to say that, of all attacked during the rise and culmination of the epidemic, at least 80 per cent. died. After the fury of its onslaught was somewhat abated, the mortuary rate fell to about 40 per cent. Even a cursory reference to topographical, meteorological and climatic influences, as pathogenic factors, would cover more space than I could hope to occupy. Much might be said, under each of these heads, of great interest and importance. I must confine my remarks, however, to the noteworthy fact, that frequent thunder, vivid flashes of lightning, and heavy rainfalls occurred during the fiercest prevalence of the epidemic. When not cloudy, the solar heat was intense, and though the nights were comparatively pleasant, the mercury rose each day to 96°, 98° and even 100° in the best protected places. The much hackneyed *malaria*—so convenient a mask for ignorance and empiricism—is pandemic, and well-nigh ubiquitous in this valley and neighboring hill-country ; yet its role in this epidemic was so inconspicuous as to be scarcely recognizable.

We are able to say but little under the head of Etiology, whatever the predisposing cause or causes ; there can be no reasonable doubt that a *specific pcison* of most deadly virulence caused the sudden and simultaneous prostration of such large numbers. No age, nor sex, nor race, nor social position afforded the least immunity. The infant at the breast, the octogenarian, and all the intermediate ages, were alike invaded. The inmates of the hovel, with its squalid poverty ; the occupants of the mansion, with their abundance, and all classes between these extremes, were attacked without discrimination. The negroes appeared to suffer a higher rate of mortality ; but this was probably owing to the impossibility of their obtaining medical attendance. The physical effect of the scourge was appalling—some fleeing in undisguised terror, and others so demoralized by panic as to be almost incapable of rendering assistance in nursing the sick. The physicians, taxed to the utmost limit of endurance, both physically and mentally, with rare and unimportant exceptions, stood heroically in solid phalanx at the post of duty, and fought this new and terrible disease, in the very teeth of defeat and death, to the bitter end ; with self-sacrificing devotion to their noble calling and the cause of suffering humanity, whilst Death held high carnival around them, and all their struggles seemed destined to defeat ; with a courage and intrepidity equal to that which prompts the soldier to face a blazing battery on the field of carnage, they never once quailed or swerved a hair's breadth from the direct line of duty. This passing tribute to professional merit may seem irrelevant in this connection, but those on whom it is bestowed most nobly earned it.

Is it catching ? Is it contagious ? were questions often propounded. I always and unhesitatingly replied, Yes ! and I believe my confreres, without a dissenting voice, agreed with me. The specific disease germ—the toxic agent—the *materies morbi*, be it bacteria, micrococci or spores ; be it a living active organism or a gaseous imponderable virus, too subtle for the lens of the microscopist or the reagents of the laboratory ; be it, in fine, what it may, is as much a *specific virus* as is that which produces small-pox, measles, scarlatina or any of the exanthemata. Whilst we are not prepared to maintain that this epidemic of dysentery was contagious in the same sense and to the same extent as the exanthematic diseases above named, we do contend that it was communicable from the sick to the well by more or less prolonged contact ; by inhaling the effluvia from the body, breath or bed of a patient, and especially from the alvine dejecta.

From close observation of the clinical history of this scourge, and from facts collected during its prevalence, we believe its contagion depended upon a *specific poison*, or toxemia, wholly independent of idio-miasmata or *crowd poison*, so strongly emphasized as a fertile source of propagation by the learned and highly distinguished Dr. Joseph J. Woodward, of the U. S. Army, in his classical work on the Medical and Surgical History of the War. Did time and space permit, we could bring an array of facts in support of our views on this point, which, we think, would be conclusive and irrefutable; but even a cursory reference to the data we possess on this subject would lead us quite beyond reasonable limits, and (however regretfully) we must forego the task.

Having thus briefly and roughly sketched the outlines of this epidemic, we may conclude with a brief reference to the important subject of

TREATMENT.

The usual dose of salts and laudanum or paregoric, by which the treatment is begun, was found, after repeated trials, to be a failure in its effects, and was abandoned; hypodermic injections of morphia sulph., $\frac{1}{8}$ to $\frac{1}{4}$ grain, supplemented by large cataplasms of mustard to stomach and bowels, and repeated as occasion required, afforded relief to the nausea and vomiting, and thus added to the comfort of the sufferer; irrigation of the rectum and colon with large enemata of *hot* water, carried by a gum catheter attached to a Davidson syringe above the sigmoid flexure and pumped into the bowels very slowly, in large quantity, at intervals of two, four or six hours, afforded great comfort, and in many ways was thought salutary. It was soothing to the hyperæmic and hyperæsthetic mucosa, and possesses, when thus intelligently applied hæmostatic, antiseptic and calminative properties of great value. By removing the semi-putrescent *debris*, consequent upon the morbid condition of the parts, it is reasonable to conclude that not only a fruitful source of irritation is got rid of, but a probable source of auto-infection also; hence I cannot insist too strongly on copious and frequently repeated irrigation, as one of the most valuable remedial measures at our command in the treatment of all severe types of dysentery. It possesses the additional advantage of being absolutely harmless, when properly used, and is often a source of unspeakable comfort to the sufferer. Although at variance with very high authority, I must insist on *hot* water—as hot as can be borne—in preference to cold, iced or tepid, and this preference has been determined by repeated clinical observations of the effects produced by enemata at different temperatures. After thorough irrigation, the water having passed off, it was my custom to introduce a suppository of solid opium, made soft by glycerine or water, high above the sphincter, and retain it there, if necessary, for an hour or more by a suitable compress pressed firmly by careful hands against the parts. The quantity of opium thus used varied, from 1 to 8 or 10 grains, according to age and other peculiarities of the patient. The prostration, and tendency to cardiac failure, required an early resort to measures of support; brandy, whisky, milk punch, egg-nogg, meat juices, hypodermics of fluid extract digitalis, etc., were the means employed for this purpose, and which appeared to do most good.

Heroic doses of calomel, advocated by some, were found to be useless, if not pernicious. Small alterative doses of mercury (sub. mur.) in fractions of a grain, repeated every four to six hours, seemed to do good in some eases, by moistening the tongue, and other evidences of emulging the secretions; but its warmest advocates could claim no marked curative effects for mercury administered either tentatively or heroically. This fact, added to popular prejudice, caused it to be abandoned. Ipecacuanha, in doses of 20 to 40 grains, well mixed with fl. ʒ i or ij of syrup of orange, always did good service, and no doubt was the means of saving some lives. The writer administered it in the following manner: Thirty minutes or more before giving the drug, the abdomen, from ensiform cartilage, was covered with a mustard cataplasm wide enough to embrace the entire surface, and 20 to 30 gtts. tincture opii given. When the pungency of the mustard can be endured no longer, it is removed, and the bolus of ipecac washed down with a single swallow of water. The patient is required to lie flat on his back, not move or speak if possible to avoid it, and to swallow absolutely nothing, for three hours. Any time during these three hours, if restlessness and complaint are at all troublesome a hypodermic of $\frac{1}{4}$ to $\frac{1}{2}$ grain of morphia is administered. This will generally restore quiet, and prevent premature vomiting. Three hours after the ipecac, small pellets of ice may be swallowed, and a few spoonfulls of boiled milk may be given occasionally. If prostration is much increased by the relaxation, nausea, etc., caused by the ipecac, milk punch, toddy, and, if deemed necessary, hypodermics of whisky, brandy, ether, etc., may be used to support the circulation. The first dose of ipecac rarely fails to produce feculent stools, with less blood and mucous, free diaphoresis, and great amelioration of all the symptoms. In ten to twelve hours this course may be repeated, the dose of ipecac being a little smaller. The ipecac being slightly diminished in quantity, may be thus repeated at intervals of ten or twelve hours until three or four doses are used, giving supporting fluids cautiously in the intervals between the doses. If no marked good impression is made by this drug, thus used, in forty-eight hours it should be abandoned. The faithful use of irrigation, as above indicated, and the ipecac treatment fairly tried, and each having failed (and fail they did In many cases), the important question presents itself, What next? I answer, unhesitatingly, *Opium—Brandy!* The remarkable insusceptibility of the system to the physiological effecis of opium was a distinctly marked feature of this epidemic. This insusceptibility we regard as an indication to push the drug, regardless of quantity until its physiological effects are produced.

To illustrate, I will briefly refer to a case: A gentleman, fifty years old, presenting a typical case, had had saline purgatives, thorough irrigation with copious hot water enemata, frequently repeated; had a 40-grain dose of ipecac ten hours subsequently, a 30-grain dose, and twelve hours thereafter another dose of 20 grs. The dysenteric symptoms were but slightly ameliorated, and the subjective symptoms forbade the further use of ipecac. A suppository of 10 grains of solid opium, properly softened, was passed high up into the rectum and retained by compress to nates; 2 grs. opii in pill form were administered per ovem every two hours for twenty-four hours, the suppository of 10 grains being repeated every twelve hours, and a hypodermic of $\frac{1}{2}$ grain sul. morphia used six hours after each suppository. Thus, he took 24 grains of solid opium of excellent quality per ovem, 30 grains by suppository, and $1\frac{1}{2}$ grains of sulph. morphine hypodermically, within twenty-four hours. The suppositories were retained and presumably absorbed, and no emesis having occurred, the 24 grains taken into the stomach were also presumably appropriated; and yet this man, perfectly free from all suspicion of opium-habit, had not the slightest symptom of narcotism or any other unpleasant symptom which could be attributed to opium. It is proper to state that whilst this heroic plan was being pursued, the patient was constantly watched with the view of detecting the first indications of opium-poisoning, but none appeared, not even marked contraction of pupils. The patient being greatly prostrated, with distinct tendency to cardiac failure, had the opium treatment supplemented vigorously with egg-nogg, milk punch, and hypodermics of fluid extract of digitalis. The opium treatment was continued in gradually diminished doses at progressively increasing intervals, with brandy and other supports, for several days, when he slowly emerged from the very jaws of death and entered upon a tedious but perfect convalescence. During all this time no symptom of opium narcosis, at any time, appeared.

A few years previous to this epidemic the writer had occasion to treat several cases of sporadic dysentery in this same family, and the lady—wife of case just stated—acquired great confidence in, and partiality for, a mixture then used by me, under the name of "Creasote Mixture," composed of 4 grains sulph. morphine, 32 gtts. creasote, and 4 ounces of camphor water, and directed in teaspoonful (ʒ i) doses after each disturbance of the bowels, etc. Immediately after this epidemic began she requested me to prepare for her half a pint (℥ 8) of the mixture, which I did. When it was delivered to her, three or four of the family were prostrated with, and under treatment for, flux. The lady, after losing several night's sleep and suffering great anxiety and alarm, was seized early one morning with the symptoms of the disease in a violent form; she went to her bottle of creasote mixture and, in the presence of a grown daughter, poured out and drank over half a teacupful (about 3 ounces), or 3 grains of morphine, 24 drops of creasote, and 3 ounces of camphor water! Previously she had vomited and was much disturbed with her bowels. Within an hour after swallowing the draught she was found asleep in a back room by some of the family; nothing was said to the physicians about what she had done. In the confusion and anxiety incident to the care of several other bad cases in the house, she was forgotten and no atten paid her for several hours, when she was found fast asleep and sweatina profusely. As respiration was free from stertor, regular and not very much slower than normal, no means seemed necessary to combat the effects of the morphia, and she was let alone. She continued to sleep for twelve hours; sweated profusely, and awoke feeling hungry. All symptoms of dysentery were gone, and she passed through the epidemic of several weeks' duration, exposed day and night to the effluvia from the bodies of the sick, dying and dead, losing sleep and suffering inexpressible distress and anxicty of mind, without having any symptom of the prevailing scourge afterward.

It would be easy to add many cases illustrative of the extraordinary tolerance of opium in this epidemic, but the above will suffice. An almost equal insusceptibility to the effects of alcoholic stimulants was manifest. The poison seemed to act with special virulence on the nerve centres of organic life. The cerebro-spinal system was much less affected, the intellectual faculties remaining intact to the last.

I am conscious of having done nothing more than outline the treatment of this scourge, in what has preceded. It may be briefly summarized in saying that ipecacuanha, opium and its preparations, faithful and frequent irrigation of the bowels with copious *hot* water enemata, brandy or whisky, milk and meat juices or broths, good nursing, cleanliness, and the assiduous use of deodorants and disinfectants—all used with intelligent discrimination—afforded the best results. All the systems of medicine known in civilized Christendom were brought into use by their various votaries, and *all* alike most signally failed. Many cases were moribund in a few hours after attack, and no case recovered without the best of care.

